# Cathelicidin-WA Protects Against LPS-Induced Gut Damage Through Enhancing Survival and Function of Intestinal Stem Cells

**DOI:** 10.3389/fcell.2021.685363

**Published:** 2021-07-26

**Authors:** Sisi Wang, Lixia Kai, Luoyi Zhu, Bocheng Xu, Nana Chen, Teresa G. Valencak, Yizhen Wang, Tizhong Shan

**Affiliations:** ^1^College of Animal Sciences, Zhejiang University, Hangzhou, China; ^2^Key Laboratory of Animal Feed and Nutrition of Zhejiang Province, Ministry of Education, Hangzhou, China; ^3^Zhejiang Provincial Laboratory of Feed and Animal Nutrition, Hangzhou, China

**Keywords:** CWA, intestinal stem cell, intestinal barrier, intestinal disease, regeneration

## Abstract

Preservation of intestinal stem cells (ISCs) plays a critical role in initiating epithelial regeneration after intestinal injury. Cathelicidin peptides have been shown to participate in regulating intestinal damage repair. However, it is not known how exactly Cathelicidin-WA (CWA) exert its function after tissue damage. Using a gut injury model in mice involving Lipopolysaccharide (LPS), we observed that CWA administration significantly improved intestinal barrier function, preserved ISCs survival, and augmented ISCs viability within the small intestine (SI) under LPS treatment. In addition, CWA administration effectively prevented proliferation stops and promoted the growth of isolated crypts. Mechanistically, our results show that the appearance of γH2AX was accompanied by weakened expression of SETDB1, a gene that has been reported to safeguard genome stability. Notably, we found that CWA significantly rescued the decreased expression of SETDB1 and reduced DNA damage after LPS treatment. Taken together, CWA could protect against LPS-induced gut damage through enhancing ISCs survival and function. Our results suggest that CWA may become an effective therapeutic regulator to treat intestinal diseases and infections.

## Introduction

The intestinal barrier plays an essential role in human and animal health. A functional intestinal barrier mediates nutrient absorption and controls toxins and bacteria crossing the intestinal epithelium to maintain the body’s internal milieu. Several gastrointestinal tract diseases such as pathogen infection and inflammatory bowel disease (IBD) always come along with intestinal barrier dysfunction and altered intestinal permeability (Yi et al., 2017; Schoultz and Keita, 2020). Compromised barrier function leads to a systematic influx of microbial products and enhanced enteric infection (Thaiss et al., 2018). Thus, for knowing how to regulate the intestinal barrier and function at different conditions, including intestinal diseases and bacterial infection, more and functional studies are required.

Antibiotics have been widely used to treat gastrointestinal infection and related disorders. However, the overuse of antibiotics might have given rise to bacterial resistance to drugs. Moreover, a new study has revealed that frequent use of antibiotics may be directly related to an individual’s risk for IBD, ulcerative colitis, and Crohn’s disease (Nguyen et al., 2020). Thus, it is imperative to find targeted substitutes with antimicrobial effects. Antimicrobial peptides (AMPs) are small proteins with antibacterial, antiviral, and antifungal activities. It has been proved that the biochemical properties and pharmacodynamics of AMPs enable them far more refractory to resistance evolution than traditional antibiotics (Lazzaro et al., 2020). Cathelicidin peptides, a family of AMPs, apart from their antimicrobial activity (Zhang et al., 2015), also function as immune regulators (Hancock et al., 2016). Recently, several studies have shown that cathelicidin peptides play important roles in protecting intestinal barrier integrity in infected mice (Otte et al., 2009; Han et al., 2016). Our previous studies have demonstrated that cathelicidin-WA (CWA), a novel cathelicidin peptide from snakes, could effectively improve intestinal epithelial barrier function and enhances host defense against bacterial infection (Yi et al., 2016, 2017). However, how exogenous AMPs, just like CWA, regulate intestinal barriers and function remains unclear.

Significant epithelial regeneration is essential for rescuing intestinal barrier integrity to prevent pathological bacteria invasion. Daily intestinal epithelium renewal is fueled by Lgr5^+^ intestinal stem cells (ISCs) located at the crypt base. The ISCs continuously generate rapidly proliferating transit-amplifying (TA) cells, differentiating into absorptive enterocytes or enteroendocrine, goblet, and tuft cells as they reach the villus (van der Flier and Clevers, 2009). Due to the stark luminal environment, ISCs have developed advanced mechanisms to protect themselves from being injured, including enhanced DNA repair (Hua et al., 2012; Trentesaux et al., 2020), stem cell interconversion (Takeda et al., 2011; Tian et al., 2011), or reacquisition of stemness by differentiated progenitors and even enterocyte-lineage daughters (van Es et al., 2012; Buczacki et al., 2013; Tetteh et al., 2016; Jadhav et al., 2017; Schmitt et al., 2018). In addition, Paneth cells intermingled with Lgr5^+^ ISCs are important constituents of the ISCs niche, which not only provide essential signals supporting Lgr5^+^ ISCs for normal epithelial maintenance (Sato et al., 2011; Farin et al., 2012; Yilmaz et al., 2012) but also secrete AMPs that help preserve stem cell viability by protecting crypt from bacterial overgrowth (Ayabe et al., 2000; Bevins and Salzman, 2011). However, whether and how exogenous AMPs, like CWA, influence ISCs function during small intestine (SI) damage remains unclear. It has been reported that genome stability is crucial for ensuring normal cell proliferation and differentiation (Fuchs, 2009). SETDB1 is a histone methyltransferase that mediates the trimethylation of histone H3 at lysine9, and can safeguard genome stability (Cuellar et al., 2017). A recent study has shown that the loss of SETDB1 leads to genome instability and ISCs death (Wang et al., 2020). Emerging evidence shows that LPS is involved in inducing DNA damage (Jaiswal et al., 2009; Qiao et al., 2019). However, whether CWA could protect against the LPS-induced disfunction of ISCs *via* regulating genome instability is completely unknown.

In this study, we generated an LPS-induced internal barrier injury mouse model and determined the effects of CWA on ISCs function and mechanisms during intestinal infection. We hypothesized that CWA could preserve ISCs’ survival, enhance barrier function and accelerate regeneration *via* regulating ISCs function. Our results may reveal CWA’s novel role on ISCs and provide useful information for treating intestinal barrier dysfunction and related diseases and infections.

## Materials and Methods

### Preparation of CWA

Cathelicidin-WA was synthesized and purified (≥95%) by using a standard solid-phase method using Automatic Peptide Synthesizer (Aapptec, Louisville, KY, United States) from C-terminus to N-terminus according to the sequence designed by our laboratory (Yi et al., 2017). The peptide was prepared in sterile saline and was stored at −80°C until use.

### Animals and Experimental Protocol

**FIGURE 1:**
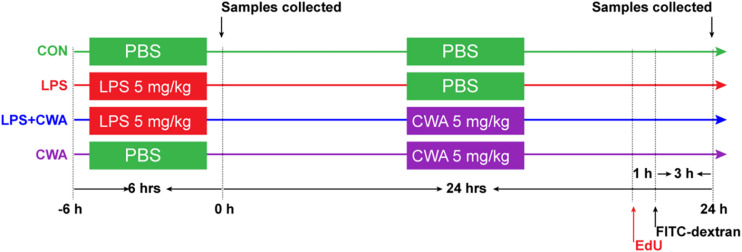
Experimental design and scheme of the animal treatments.

All animal procedures were in agreement with the Guide for the Care and Use of Laboratory Animals in Zhejiang University. The ethical committee number for the study is ZJU20170466. Lgr5-EGFP-IRES-CreERT2 reporter mice were a kind gift from the Yeguang Chen lab (Qi et al., 2017) and were maintained on a C57BL/6 background. All experimental procedures were performed under approval from Zhejiang University adhering to their regulations on care and use of laboratory animals. As shown in Figure 1, 8-weeks-old Lgr5^–^EGFP-IRES-CreERT2 reporter mice were randomly divided into four groups: in the Control and CWA group, mice were intraperitoneally injected with PBS for 6 h, followed by injection of PBS (Control) or 5 mg/kg CWA (CWA group) for 24 h; in the LPS and LPS + CWA group, mice were intraperitoneally injected with 5 mg/kg LPS (Sigma-Aldrich, L3024) for 6 h, followed by injection of PBS (LPS group) or 5 mg/kg CWA (LPS + CWA group) for 24 h.

### Isolation of Intestinal Crypts and Organoids Culture

Intestinal crypts were isolated as previously described (Sato et al., 2009). Briefly, isolated intestines were flushed with cold PBS and opened longitudinally, and further washed with cold PBS. Then, villi were carefully scraped away and small pieces (5 mm) of intestine were incubated in 2 mM EDTA in PBS for 30 min on ice. After removal of EDTA medium, the fragments were washed with PBS. After removal of PBS, fragments were poured into sterile Petri dishes and squeezed with a slide. Then, fragments were vigorously suspended with 1-ml pipette in PBS and passed through a 70-μm cell strainer (BD Bioscience). Isolated crypts were centrifuged at 150–200 *g* for 3 min. The final pure crypts were then resuspended with Matrigel (Corning, 354230) and seeded on 48-well or 24-well plates. After polymerization, crypt culture medium (Stemcell Technologies, Cambridge, MA, United States) supplemented with penicillin-streptomycin (100 U/ml) was added and refreshed every 2–3 days.

### Immunofluorescence and Immunohistochemistry

For immunofluorescence, isolated intestine samples were gently flushed with cold 4% PFA and fixed in 4% PFA for at least an hour at room temperature. Then, samples were washed with PBS three times and cryo-protected in 30% sucrose overnight at 4°C. Samples were embedded in OCT (optimal cutting temperature compound) and frozen at −80°C until usage. 5 μm sections were prepared with Cryotome FSE (ThermoFisher), then permeabilized with 0.1% Triton X-100 for 15 min at 4°C, followed by being washed three times with PBS and were then incubated for 1h in 3% BSA in PBS. Sections were then incubated overnight with the primary antibody. The following primary antibodies were used: rabbit anti-ZO-1 (Proteintech, 21773-1-AP, 1:100); rabbit anti-E-cadherin (Proteintech, 20874-1-AP, 1:300); rabbit anti-Lysozyme (Abcam, EPR2994, 1:500); rabbit anti-Ki67 (Abcam, ab15580, 1:500); rabbit anti-Clusterin (Abcam, ab230150, 1:300); rabbit anti-H2A.X (CST, 9718, 1:400). The samples were then incubated with fluorescein-labeled secondary antibodies (Abcam, ab150117, 1:300) at room temperature for 1h. DAPI (Boster, AR1176) was used for nuclei. For EdU assay, EdU powder (Ribobio, C00053) was dissolved in PBS to 1 mg/ml. EdU staining was performed according to the manufacturer’s instruction (Beyotime Biotechnology, C0078S). For immunohistochemistry, samples were washed, paraffin-embedded and sectioned. Paraffin sections were deparaffinized and then antigen-retrieved in 10 mM sodium citrate, pH = 6.0. Sections were then blocked with 3% (w/v) BSA and 0.1% (v/v) Triton X-100 for 1 h after endogenous peroxidase quenched by 3% H2O2. Samples were incubated over-night at 4°C with rabbit anti-Olfm4 (CST, 39141, 1:500) and rabbit anti-SETDB1 (CST, 93212, 1:1,000). Then samples were washed with PBS and incubated with horseradish peroxidase (HRP)-conjugated secondary antibodies (Huabio, HA1001-100, 1:100) for 1 h at room temperature. DAB (Solarbio, DA1010) was then applied for the color reaction according to the manufacturer’s instructions. For TUNEL staining, a TUNEL kit (Beyotime Biotechnology, C1091) was used according to the manufacturer’s instructions.

### Gut Permeability Assessment

D-LA detection: Serum concentration of D-LA was assessed by using commercial mouse ELISA kits (Nanjing JianCheng, Nanjing, China) according to the manufacturer’s instructions. FITC-dextran detection: 4 kDa fluorescein isothiocyanate (FITC)-dextran (Sigma-Aldrich, St. Louis. MO, United States) was dissolved in PBS to a concentration of 40 mg/ml. Mice were anesthetized 3 h following gavage with dextran (10 mg/kg) and blood was collected in a dark environment. Serum was collected by centrifuging (1,500 rpm, 15 min, 4°C) and fluorescence was quantified at an excitation wavelength of 485 nm and an emission wavelength of 535 nm.

### RT-qPCR

Total RNA was extracted from purified crypts using trizol reagent (Invitrogen, United States) and reverse transcribed into cDNA with the GoScript Reverse Transcription System (Promega, Madison, WI, United States). Amplification was performed with SYBR Green master mix (Roche, Mannheim, Germany) using a StepOnePlus^TM^ Real Time PCR systems (Applied Biosystems, Foster City, CA, United States). The gene-specific primers for the q-PCR are listed in Table 1 and the relative mRNA expression of the target gene was determined using the 2^–ΔΔCt^ method.

**TABLE 1:** The gene-specific primers for the q-PCR.

**Target genes**	**Forward (5′–3′)**	**Reverse (5′–3′)**
Lgr5	CGGGACCTTGAAGATTTCCT	GATTCGGATCAGCCAGCTAC
Olfm4	CGAGACTATCGGATTCGCTATG	TTGTAGGCAGCCAGAGGGAG
Tnfrsf19	CTTCCGTGACAGCATTTACCTT	CTGCTCAGTGAAGCCATAGGG
Smoc2	CCCAAGCTCCCCTCAGAAG	GCCACACACCTGGACACAT
Wnt3	GGGCCAGCAGTACACATCTC	TCACACCTTCTGCTACGCTG
β-catenin	TGGACGTGGGCGAACTTTTA	GCGTTCTCGAGGACCAGTTT
Ascl2	GCCTGACCAAATGCCAAGTG	ATTTCCAAGTCCTGATGCTGC
Axin2	GCTCCAGAAGATCACAAAGAGC	AGCTTTGAGCCTTCAGCATC
Cd44	CCTTGGCCACCACTCCTAAT	TGGGAGTCTTCACTTGGGGTA
EphB2	TCTATGTCTTCCAGGTGCGG	TGGTCTGGTACTCGGCTTCT
Hey1	AACGACATCGTCCCAGGTTT	ATTGATTCGGTCTCGTCGGC
Hes1	TCAACACGACACCGGACAAA	ATTCTTGCCCTTCGCCTCTT
Dll1	GGTTTGTGTGTGACGAGCAC	ATCTTCTCCCCTCTGTCCCC
Dll4	CCAGCAACCCCTGTCGAAAT	ACAGTGCTGGCCATAGTAGC
Reg3b	ACTCCCTGAAGAATATACCCTCC	CGCTATTGAGCACAGATACGAG
Reg3g	ATGCTTCCCCGTATAACCATCA	GGCCATATCTGCATCATACCAG

### Western Blot

Total proteins of whole intestinal tissue were extracted using Protein Extraction Reagent (KeyGENBioTECH, Nanjing, China) and the concentrations were determined with the BCA Assay Kit (KeyGENBioTECH, Nanjing, China). Equal amounts of protein were separated by SDS-PAGE and electroblotted onto polyvinylidenedifluoride membranes (PVDF) (Millipore, Bedford, MA, United States) followed by blocking with 5% fat-free milk. Then, the membranes were probed separately with rabbit anti-villin (Proteintech, 16488-1-AP, 1:2,000), anti-E-cadherin (Proteintech, 20874-1-AP, 1:2,000), anti-ZO-1 (Proteintech, 21773-1-AP, 1:2,000), anti-Cleaved Caspase-3 (CST, 9664, 1:1,000), anti-SETDB1 (CST, 93212, 1:1,000) and anti-H2AX (CST, 9718, 1:1,000) overnight at 4°C. After washing with TBST, the membranes were incubated with goat anti-rabbit secondary antibodies (Huabio, HA1001-100, 1:2,500) for 1 h at room temperature. Signals were detected using ChemiScope3500 Mini System, after that ECL was added onto the membranes and band intensity was quantified by Image J software.

### Statistical Analysis

Data are presented as means ± SD. Comparisons between two groups were performed using a two-sided, unpaired *t*-test. One-way ANOVA with Duncan’s multiple range test was used for comparison between multiple Groups. *P* < 0.05 was considered significant.

## Results

### CWA Protects Against LPS Induced-Gut Injury and Animal Death

**FIGURE 2:**
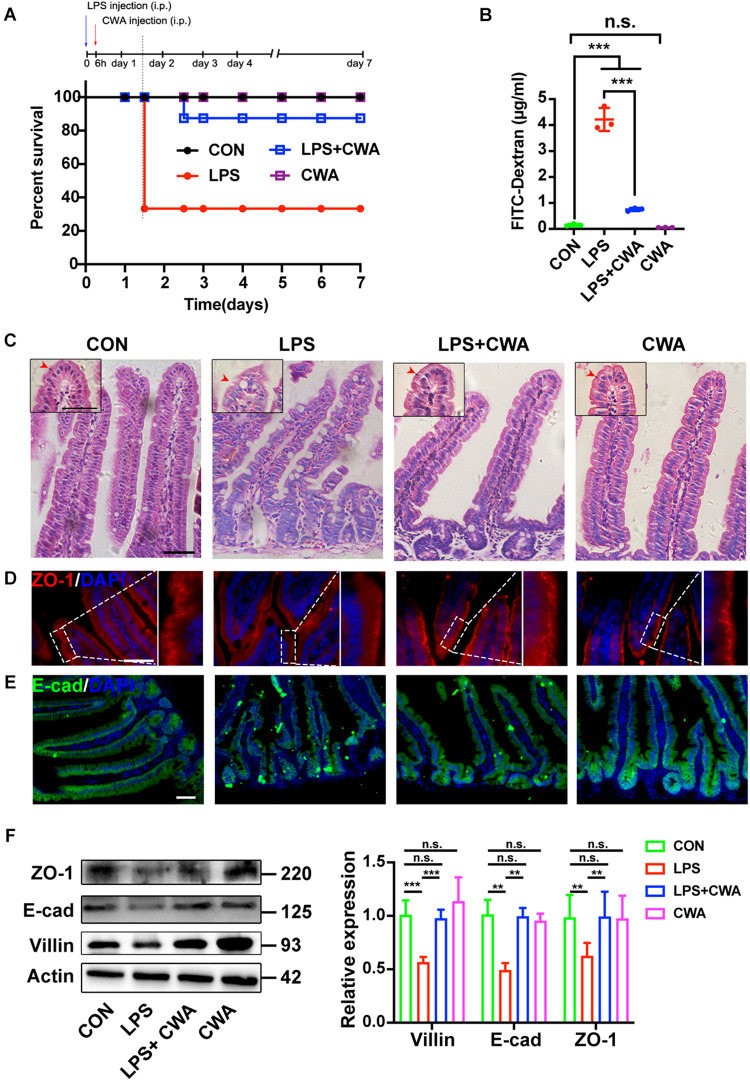
CWA protects against LPS-induced gut injury and animal death. **(A)** Survival was monitored daily for 7 days. (*n* = 10 mice). **(B)** Gut permeability was determined by FITC-dextran. FITC (fluorescein isothiocyanate)-dextran recovered from the serum 3 h after oral gavage. **(C)** H&E images of villous structure, the red border outside the villi indicates brush border which is composed by microvilli. Boxed region indicates magnified view of microvilli. red arrows indicate length of microvilli. **(D,E)** Representative images of ZO-1 **(D)** and E-cadherin **(E)** staining in thin sections of jejunum. **(F)** Western blot analysis of ZO-1, E-cad, and villin expression in small intestinal tissues. Scale bars, 50 μm. ***P < 0.001, **p < 0.01 by two-sided, unpaired *t*-test. All data represent at least three independent experiments.

We generated an intestinal barrier injury mouse model by intraperitoneal (IP) injection of LPS. Severe diarrhea was observed in the LPS group from 6 h after LPS IP-injection (Supplementary Figure 1). H&E results showed that the SI mucosa of LPS-treated mice exhibited severe villous atrophy (Supplementary Figure 2A). In addition, the D-LA levels were significantly increased in the LPS group compared to the control (CON) group (Supplementary Figure 2B), suggesting increased intestinal permeability from 6 h post LPS injection. To determine the role of CWA, we treated mice with CWA at 6 h after LPS injection and mice were monitored for another 7 days after CWA treatment for survival index study (Figure 2A). We found that about seventy percent of mice treated with LPS died whereas only ten percent of LPS-treated mice followed by CWA administration died (Figure 2A). Specifically, a single dose with CWA did not affect mice survival index (Figure 2A). Due to the high mortality between 24 and 36 h caused by LPS stimulation, we set out to examine what happened within the SI 24 h after CWA administration. Notably, CWA effectively improved the intestinal permeability detected using FITC-dextran (Figure 2B). H&E staining was done to visualize the brush border (Figure 2C). We found that the brush border was thin, rough and could hardly be recognized in the LPS group. In contrast, the brush border was thick, smooth and visible in the LPS + CWA group. The observation was consistent with the enhanced expression of villin protein in the LPS + CWA group compared with LPS group (Figure 2F). Furthermore, the expression of tight junction protein ZO-1 (Figures 2D,F) and adherens junction protein E-cad (Figures 2E,F) was markedly enhanced by CWA administration. Taken together, these results demonstrate that CWA administration notably mitigated LPS-induced gut injury, promoted epithelial differentiation, and increased animal survival.

### CWA Protects Lgr5^+^ ISCs From LPS-Induced Ablation

**FIGURE 3:**
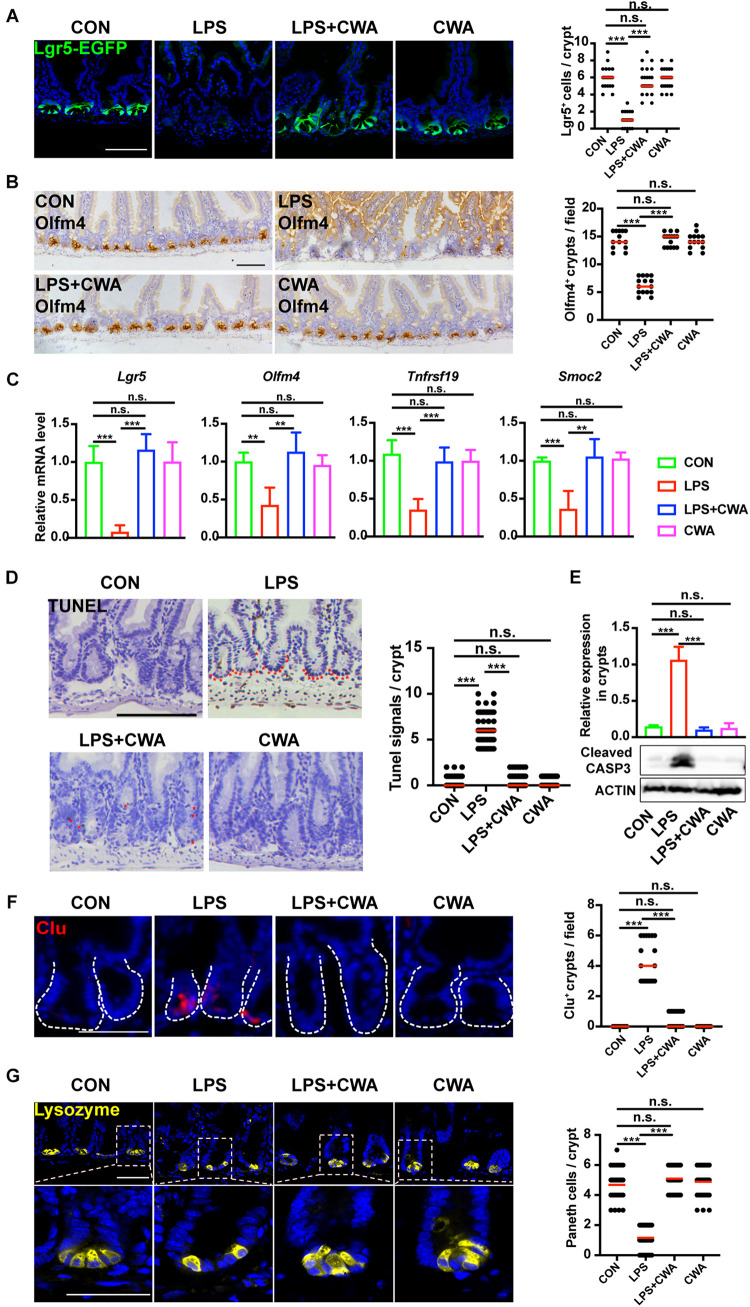
CWA protects Lgr5^+^ ISCs from LPS-induced ablation. **(A)** Representative confocal microscopy images of EGFP positive cells and numbers of Lgr5 positive cells per crypt. Scale bars, 100 μm. **(B)** IHC analysis of ISCs marker Olfm4 expression in jejunum crypts and quantification of the Olfm4 positive crypts. Scale bars, 100 μm. **(C)** Quantitative RT-qPCR analysis of *Lgr5*, *Olfm4*, *Tnfrsf19*, and *Smoc2* expression in intestinal crypts **(D)** Representative images of TUNEL assay. Red stars indicate apoptotic stem cells and number of TUNEL^+^ cells in crypts. Scale bars, 100 μm. **(E)** Western blot analysis of Cleaved Caspase-3 expression in small intestinal crypts. **(F)** Representative immunofluorescence images of Clu expression in crypt and quantification of the Clu positive crypts. Scale bars, 100 μm. **(D)** Representative confocal images of Paneth cells (Lyz+) in SI crypts and quantification of Paneth cells in each crypt. Scale bars, 50 μm. **p<0.01, ***P < 0.001 by two-sided, unpaired *t*-test. All data represent at least three independent experiments.

Given the critical role of Lgr5^+^ ISCs in restoring epithelial barrier integrity (Metcalfe et al., 2014), we next investigated the effect of CWA on Lgr5^+^ ISCs by using Lgr5-EGFP-IRES-CreERT2 reporter mice. Notably, we found that EGFP-positive cells were totally depleted in the LPS group (Figure 3A). In accordance with this, the expression of Olfm4 was dramatically decreased in the LPS group while it was kept stable in the LPS + CWA groups (Figure 3B). Loss of Lgr5-expressing cells was further confirmed by downregulated expression of Lgr5^+^ISCs signature genes (Figure 3C) and was accompanied by extensive apoptosis at the base of the crypts (Figures 3D,E). By contrast, CWA treatment markedly avoided the depletion of Lgr5^+^ ISCs (Figures 3A–D). Long-term absence of Lgr5^+^ ISCs triggers continuous Clu^+^ cell production, a unique revival stem cell population for intestinal regeneration. We found that Clu^+^ cells only appeared at the crypts of the LPS-treated mice (Figure 3F), which further indicating the loss of Lgr5^+^ ISCs. Paneth cells function as niche playing important roles in regulating Lgr5^+^ ISCs. Here, we found that the number of Paneth cells was strikingly decreased in the LPS group compared with other three groups (Figure 3G). Interestingly, we found no evidence for a decreased number of Lgr5^+^ cells, Olfm4^+^ cells and Paneth cells at 6 h after LPS injection (Supplementary Figures 2C,D,G). Collectively, these findings suggest that CWA effectively protected the Lgr5^+^ ISCs from LPS-induced ablation.

### CWA Preserves ISCs Viability and Promotes Epithelial Regeneration

**FIGURE 4:**
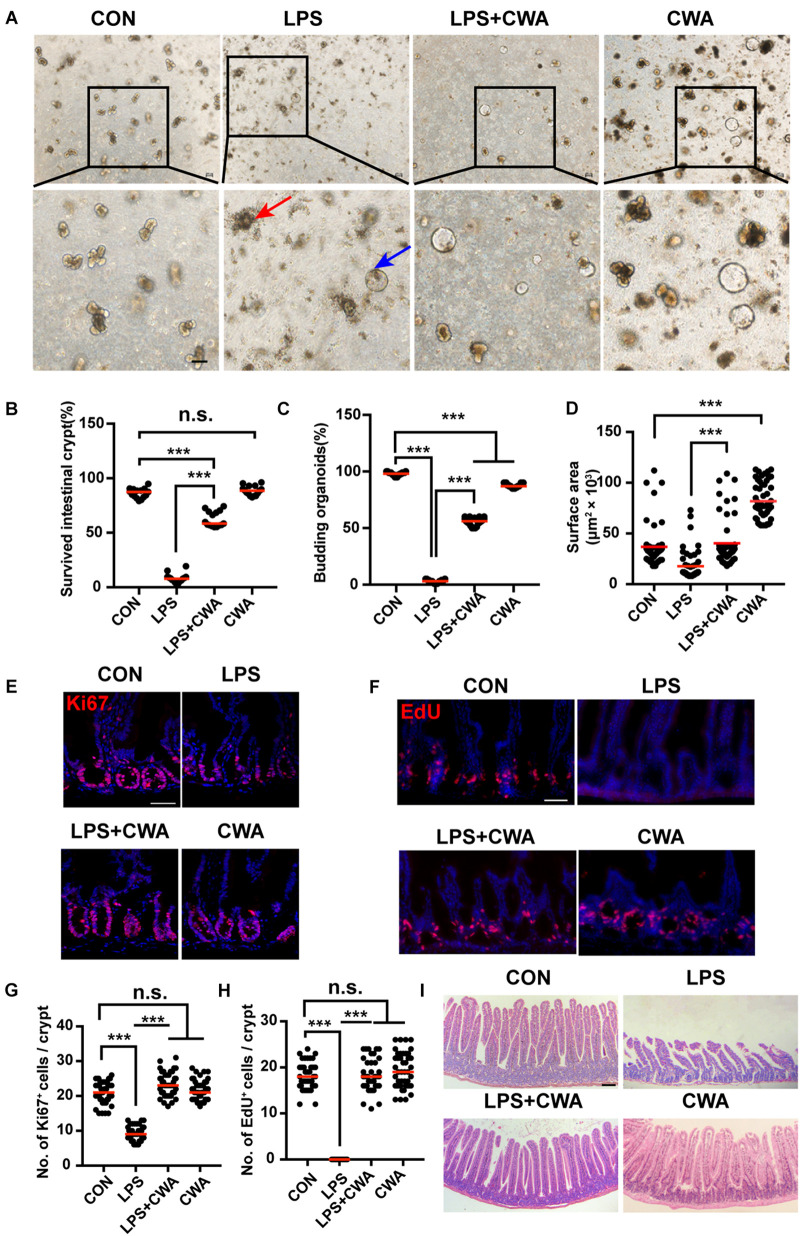
CWA preserves ISCs viability and promotes epithelial regeneration. **(A)** Representative bright-field images of crypts after 4 days of culture in ENR medium. Red arrowhead indicates collapsed organoids, blue arrowhead indicates survived organoids. Scale bars, 100 μm. **(B)** Survival rate of crypts isolated from each group. **(C)** Organoid budding, percentage of total organoids per well. **(D)** Size of SI organoids cultured in ENR medium. **(E)** Representative confocal microscopy images of Ki67 positive cells in crypts. Scale bars, 50 μm. **(F)** Representative immunofluorescence images of EdU positive cells. Scale bars, 50 μm. **(G)** Quantification of Ki67^+^ cells in each crypt. **(H)** Quantification of EdU^+^ cells in each crypt. **(I)** H&E staining of jejunum sections at 24 h after CWA administration. Scale bars, 100 μm. ***P < 0.001 by two-sided, unpaired *t*-test. All data represent at least three independent experiments.

To determine the effects of CWA on overall ISCs viability, crypts isolated from four different groups were embedded in Matrigel and cultured with a medium containing EGF, Noggin and R-spondin (ENR medium) for 3D mini-gut organoids. We found that few isolated crypts from the LPS-treated group survived (Figures 4A,B). Interestingly, these survived organoids from the LPS-treated group did not build a crypt-villus structure within 4 days in culture while organoids from other groups budded (Figure 4A). Notably, CWA treatment robustly boosted the crypt organoid-forming capacity and enhanced the size of organoids in the LPS + CWA group compared to the LPS group (Figures 4B–D). In addition, enhanced ISCs proliferation were observed in LPS + CWA group compared to LPS group, as indicated by increased Ki67 and EdU positive cells (Figures 4E–H). Notably, the EdU signals were totally disappeared in LPS group. Consistent with the increased proliferating cells in crypts, LPS + CWA mice had improved intestinal histology (Figure 4I). However, we found no change in the expression of Ki67 and EdU 6 h after LPS injection (Supplementary Figures 2E,F). Taken together, our findings demonstrate that CWA strongly enhanced ISCs viability and promoted epithelial regeneration during LPS-induced intestinal injury.

### CWA Suppresses the Genome Instability

To explore the mechanisms by which CWA improved the ISCs activity and proliferation, we first focused on Wnt and Notch pathways and performed qPCR to assess the relative gene expression of the involved genes. However, compared with the LPS treatment group, we found no evidence of enhanced expression of molecules in the Wnt/β-catenin and Notch pathway within crypts isolated from the LPS + CWA group (Supplementary Figures 3A,B). Furthermore, there were no differences in expression of the innate antimicrobial molecules *Reg3b and Reg3g* between the LPS-treated and the CWA-treated groups (Supplementary Figure 3C). Taken together, these findings imply that Notch, Wnt/β-catenin pathways may not play a major role in the treatment of LPS-induced intestinal injury by CWA.

**FIGURE 5:**
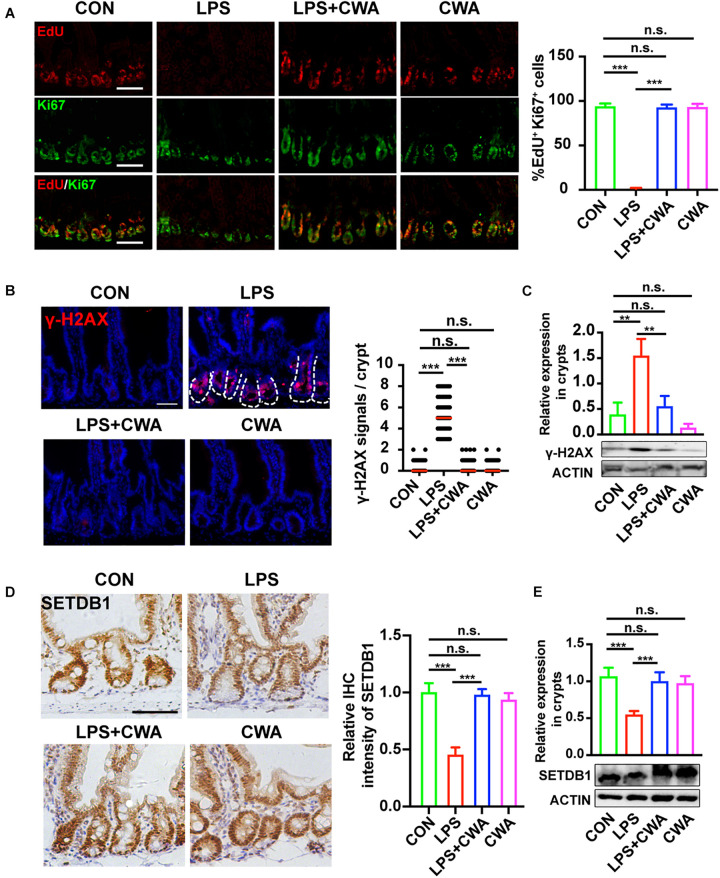
CWA suppresses the genome instability. **(A)** Representative image of co-stained EdU and Ki67 in SI crypts. EdU was allowed to incorporate for 4 h before harvest. The graph in the right is the statistical analysis of overlapped EdU and Ki67 cells. **(B)** Representative immunofluorescence images of γ-H2AX expression in crypt at 24 h after CWA administration and numbers of γH2AX + cells in crypts. **(C)** Western blot analysis of γ-H2AX expression in crypts. **(D)** Representative IHC image of SETDB1 expression at 24 h after CWA administration and analysis of SETDB1 IHC intensity. **(E)** Western blot analysis of SETDB1 expression in crypts. Scale bars, 100 μm. ***P < 0.001, **p < 0.01 by two-sided, unpaired *t*-test. All data represent at least three independent experiments.

Intriguingly, we have found that the EdU signals were completely undetectable in the late stage of the LPS group while the Ki67^+^ cells didn’t totally disappear. To further investigate the relationship between EdU^+^ cells and Ki67^+^ cells, co-staining was performed. It was found that all three groups, except the LPS group, had upward to 90% overlap of EdU^+^ cells and Ki67^+^ cells, indicating that proliferating cells in the LPS group can’t achieve DNA replication (Figure 5A), which implied the DNA damage. It has been reported that genome instability induced by decreased SETDB1 expression in ISCs could induce ISCs death (Wang et al., 2020). Here, we examined the genome instability by detecting DNA damage marker γH2AX. γH2AX signals were not detectable within 6 h after LPS treatment (Supplementary Figure 4A). Interestingly, SETDB1 notably decreased 6 h after LPS treatment (Supplementary Figure 4B). Furthermore, we found that the decreased expression of SETDB1 was rescued 24 h after CWA administration, whereas the LPS-treated group still exhibited reduced SETDB1 expression (Figures 5D,E). The decrease of SETDB1 was synchronous with acute expression of γH2AX while mice treated with CWA did not show γH2AX signals (Figures 5B,C). Together, our findings suggest that CWA may eliminate LPS induced DNA damage by enhancing genome stability.

## Discussion

This study demonstrates that CWA may increase overall gut health by enhancing barrier function, augmenting ISCs survival and facilitating epithelial regeneration in LPS-induced gut injury. Strikingly, we can show for the first time that CWA prevented ISCs’ death and apoptosis by being involved in regulating genome stability. Our findings provide insight into the regulatory role of CWA in regulating intestinal barrier and ISCs function, and it could be used as a therapeutical target to treat related intestinal diseases and bacterial infections.

Our data show that LPS treatment led to the ablation of Lgr5^+^ISCs in the late stage after LPS injection. However, the number and proliferative function of Lgr5^+^ISCs didn’t change in the early stage (6 h) after LPS injection when the intestinal epithelium has been disrupted. The delayed damage to the ISCs may benefit from the intestinal structure. The crypt developed into a highly protective environment that supports proliferation and minimizes damage to stem cells. Numerous studies have confirmed this protective effect of crypts (Trezise et al., 1992; Liu et al., 2012; Kaiko et al., 2016). The kinetics of the response of SI epithelial barrier to LPS stimulation have been well documented (Lai et al., 2013; Williams et al., 2013; Han et al., 2016; Zong et al., 2016, 2019), so we didn’t detect the barrier function in more detail in the early stage after LPS injection.

The most active cycling Lgr5^+^ ISCs are dividing approximately every 24 h (van der Flier and Clevers, 2009). In our study, since the CWA treatment time was only maintained for 24 h, the Lgr5^+^ ISCs could not experience death and reproduce during such short period. Thus, we conclude that CWA treatment in our model prevented LPS-induced Lgr5^+^ ISCs death instead of revitalizing. Lgr5^+^ ISCs are indispensable for intestinal regeneration after damage. It got evident by the failure to restore the epithelium while re-appearance of Lgr5^+^ ISCs was blocked (Metcalfe et al., 2014). Here, we provide a potential possibility that the preservation of Lgr5^+^ ISCs enabled the intestine to initiate rapid regeneration after LPS-induced gut injury. Thus, it may further prevent pathogen invasion, which contributed to host survival.

Here, we still detected the proliferative cell marker Ki67 in the crypts of the LPS group, suggesting that although Lgr5^+^ISCs were depleted, there were some other proliferative stem cells surviving in the crypts. Previous studies have shown that Lgr5^+^ISCs are more intolerant to injury compared to other stem cells (Montgomery et al., 2011; Yan et al., 2012). Acute injury results in the loss of Lgr5^+^ISCs, but leaves behind injury-resistant Paneth cell precursors, +4 stem cells and intact niches (Barker, 2014). It is possible that these stem cells, which still have proliferative activity, could be converted into Lgr5^+^ISCs and promote intestinal epithelial proliferation. Here, due to the rapid death of mice, we can’t further explore.

Wnt/β-catenin signaling is critical for ISCs function maintenance and organoid growth *ex vivo* (Reya and Clevers, 2005; Sato et al., 2009). It has been reported that activation of the TLR4 pathway mediates LPS-induced injury in enterocytes *via* impairing β-catenin signaling (Sodhi et al., 2010). In our current study, we also noticed that the Wnt pathway was altered after LPS stimulation, but not rescued after CWA treatment. In addition, single administration of CWA didn’t change the expression of genes in the Wnt/β-catenin pathway compared to the CON group. These results imply that CWA is perhaps not acting to protect ISC function through regulating the Wnt signaling pathway. It has been reported that EGF signaling inhibition halts DNA replication and induces quiescence of ISCs (Basak et al., 2017). It has also been found that the complete inhibition of EGF signaling can only lead to Lgr5^+^ ISCs death after a few days (Basak et al., 2017). However, in our study, we found a rapid loss of Lgr5^+^ ISCs (within 30 h) after LPS treatment. In addition, we didn’t find quiescence of ISCs because Ki67^+^ cells were found in crypts. These results indicated that EGF signaling may not play the main role in LPS-induced impaired proliferation.

It has been reported that LPS elicited innate AMPs secretion (Ayabe et al., 2000). In this study, we have also examined the crypts mRNA expression of innate AMPs. Expectedly, the innate AMPs molecules Reg3b and Reg3g notably increased in response to LPS stimulation regardless of CWA administration. In addition, we found no evidence for increased expression of innate AMPs in response to a single CWA administration. Our results suggest that CWA was not involved in regulating the production of innate AMPs. Thus, exogenous AMPs may prove very necessary when administered after LPS-induced intestinal injury.

A previous study has reported that SETDB1 inactivation led to ISC death (Wang et al., 2020). Interestingly, we found that although the number of Lgr5^+^ and proliferating cells didn’t decrease within 6 h after LPS injection when mice exhibited decreased expression of SETDB1. We postulate that LPS induced the decrease of SETDB1 6 h after injection or earlier, but the short-term reduction of SETDB1 did not cause ISCs cell death. We have previously found that LPS strongly stimulates oxidative stress and accumulates reactive oxygen species (ROS) in the intestine (Wu et al., 2018a,b). Moreover, it has been reported that a ROS inhibitor could recover decreased SETDB1 expression (Park et al., 2019). Additionally, we have shown that CWA could eliminate the LPS-driven ROS accumulation (Chen et al., 2018; Wu et al., 2018b). Our results suggest that immediate administration of CWA may elevate SETDB1 expression by reducing ROS, thus genome stability could have been maintained and ISCs cell death was prevented in CWA treated group. Here, we show one possible mechanism that CWA beneficially affected genome stability by enhancing SETDB1 expression. However, we can’t exclude the possibility that other factors also contribute to this process.

## Conclusion

**FIGURE 6:**
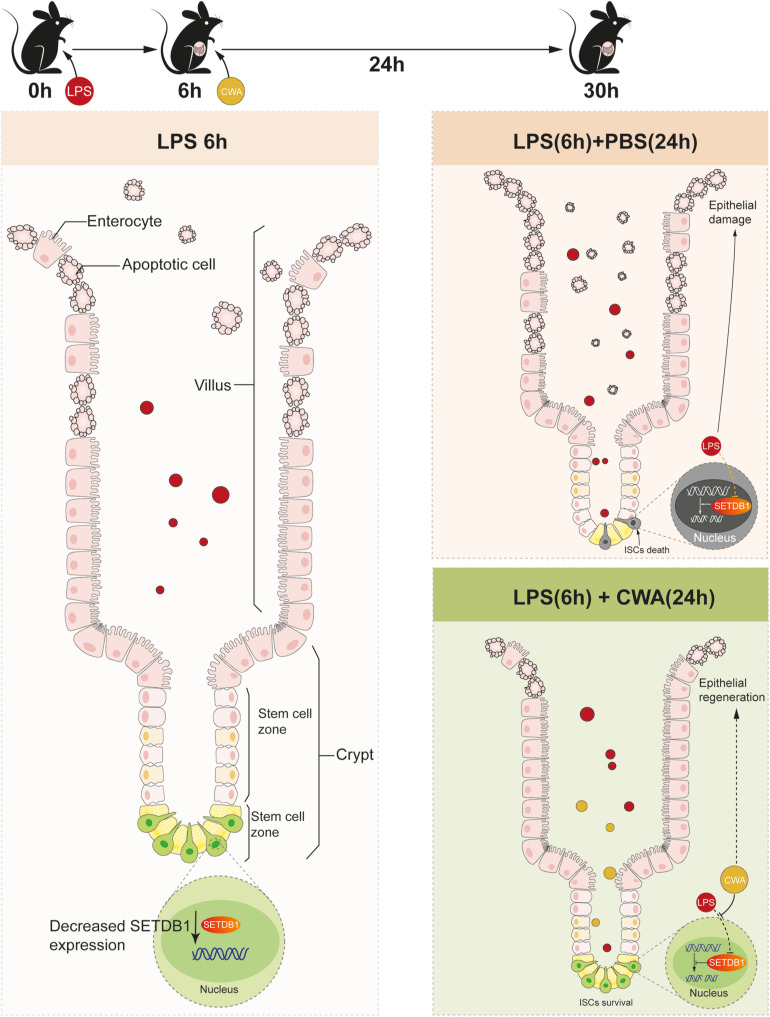
Schematic Diagram. Decreased expression of SETDB1 was found within 6 h after LPS treatment and short-term reduction of SETDB1 didn’t lead to DNA damage. CWA treatment significantly restores the decreased expression of SETDB1, thus exhibiting beneficial regulation on genome stability and preventing DNA damage. Stabilized genome enables ISCs to survive under infection conditions and promotes regeneration.

Our novel findings showed that CWA might exert beneficial effects on maintaining genome stability and function of ISCs during LPS-induced gut injury. Moreover, the preservation of ISCs guarantees rapid epithelial renewal to prevent further intestinal damage (Figure 6). Our results suggest that CWA might serve as a powerful new treatment in preventing intestinal diseases such as pathogen infection and IBD.

## Data Availability Statement

The original contributions presented in the study are included in the article/Supplementary Material, further inquiries can be directed to the corresponding author/s.

## Ethics Statement

The animal study was reviewed and approved by Care and Use of Laboratory Animals in Zhejiang University.

## Author Contributions

SW and TS contributed to conception and design of the study. SW and BX contributed to analysis and interpretation of data. LZ and LK performed the material preparation and data collection. SW wrote the first draft of the manuscript. All authors commented on previous versions of the manuscript and read and approved the final manuscript.

## Conflict of Interest

The authors declare that the research was conducted in the absence of any commercial or financial relationships that could be construed as a potential conflict of interest.

## Supplementary Material

Supplementary Figure 1 Representative image of diarrhea at 6 h after LPS injection. Severe diarrhea was observed in the LPS group from 6 h after LPS IP-injection.

Supplementary Figure 2 Responses of small intestine to LPS stimulation at 6 h after injection. **(A)** H&E staining of jejunum sections at 6 h after LPS injection. Scale bars, 100 μm. **(B)** Gut permeability was determined by D-LA at 6 h after LPS injection. **(C–G)** Representative immunofluorescence images and quantification of Lgr5^+^ cells (scale bar, 50 μm), Olfm4^+^ cells (scale bar, 100 μm), ki67^+^ cells (scale bar, 100 μm), EdU^+^ cells (scale bar, 100 μm) and Paneth cells (scale bar, 50 μm) at 6 h after LPS injection. ^∗∗∗^*P* < 0.001 by two-sided, unpaired *t*-test. All data represent at least three independent experiments.

Supplementary Figure 3 CWA promotes intestinal epithelial proliferation independent of Notch, Wnt/β-catenin signaling pathways and innate AMPs. **(A)** RT-qPCR analysis of relative crypts mRNA expression of Wnt pathway genes in crypts. **(B)** RT-qPCR analysis of relative crypts mRNA expression of Notch pathway genes in crypts. **(C)** RT-qPCR analysis for relative crypts mRNA expression of Reg3b and Reg3g innate antimicrobials in crypts. *^∗∗∗^P* < 0.001 by two-sided, unpaired *t*-test. All data represent at least three independent experiments.

Supplementary Figure 4 Immunofluorescence and IHC analysis of γ-H2AX and SETDB1. **(A)** Representative immunofluorescence images of γ-H2AX expression in crypt at 6 h after LPS injection and numbers of γ-H2AX^+^ cells in crypts at 6 h after LPS injection. **(B)** Representative IHC image of SETDB1 expression at 6 h after LPS injection and analysis of SETDB1 IHC intensity. ^∗∗∗^*P* < 0.001 by two-sided, unpaired *t*-test. All data represent at least three independent experiments.
