# Supplementary figures and images for: Cathelicidin-WA Protects Against LPS-Induced Gut Damage Through Enhancing Survival and Function of Intestinal Stem Cells
Source: Front Cell Dev Biol. 2021 Jul 26;9:685363. doi: 10.3389/fcell.2021.685363 (PMC8350165; doi:10.3389/fcell.2021.685363)

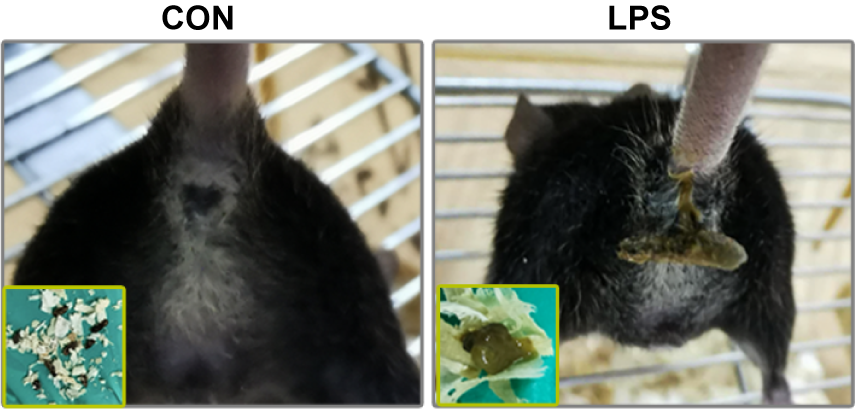

Supplement: Supplementary Figure 1 — Representative image of diarrhea at 6 h after LPS injection. Severe diarrhea was observed in the LPS group from 6 h after LPS IP-injection. [file Image_1.TIF]

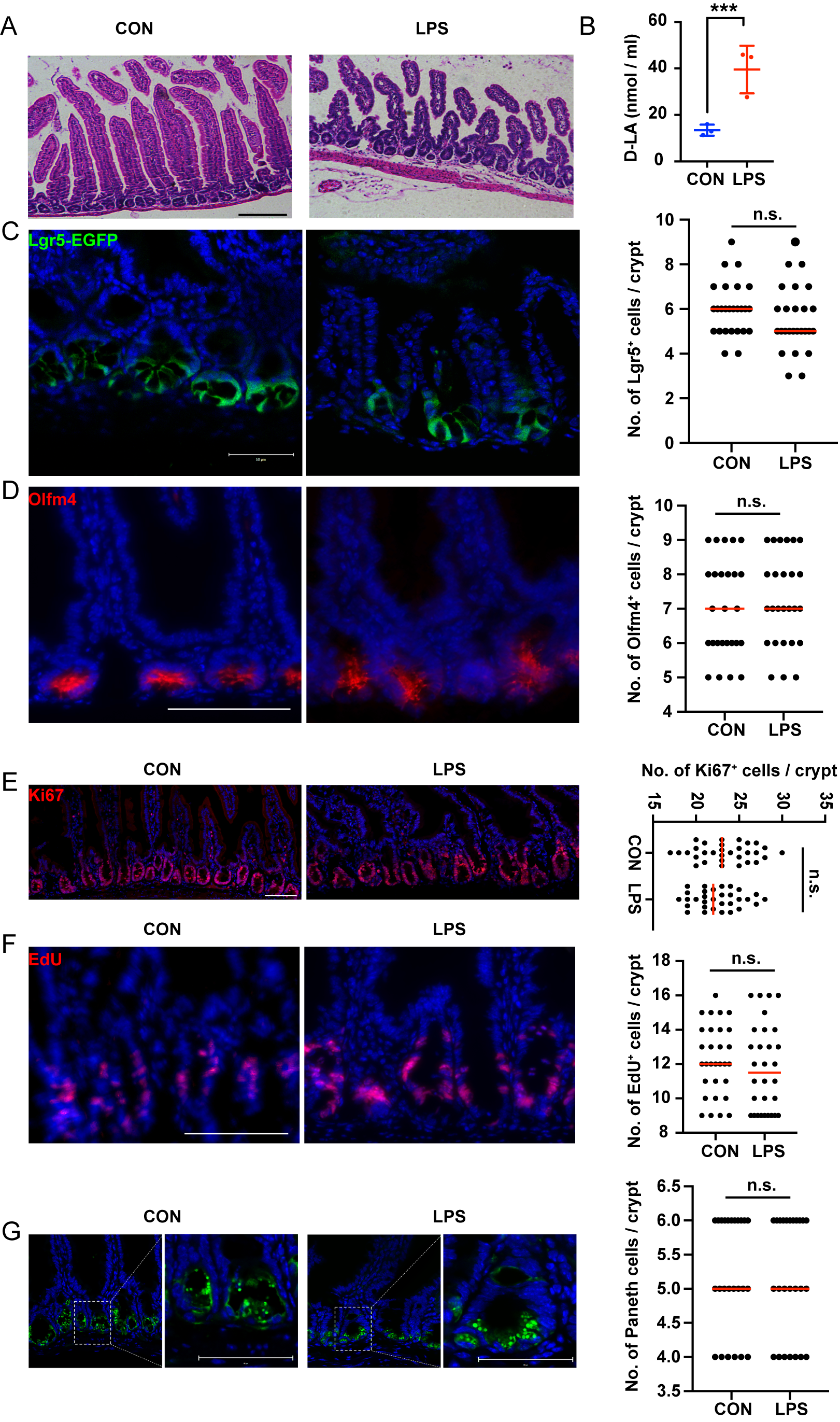

Supplement: Supplementary Figure 2 — Responses of small intestine to LPS stimulation at 6 h after injection. (A) H&E staining of jejunum sections at 6 h after LPS injection. Scale bars, 100 μm. (B) Gut permeability was determined by D-LA at 6 h after LPS injection. (C–G) Representative immunofluorescence images and quantification of Lgr5+ cells (scale bar, 50 μm), Olfm4+ cells (scale bar, 100 μm), ki67+ cells (scale bar, 100 μm), EdU+ cells (scale bar, 100 μm) and Paneth cells (scale bar, 50 μm) at 6 h after LPS injection. ∗∗∗P < 0.001 by two-sided, unpaired t-test. All data represent at least three independent experiments. [file Image_2.TIF]

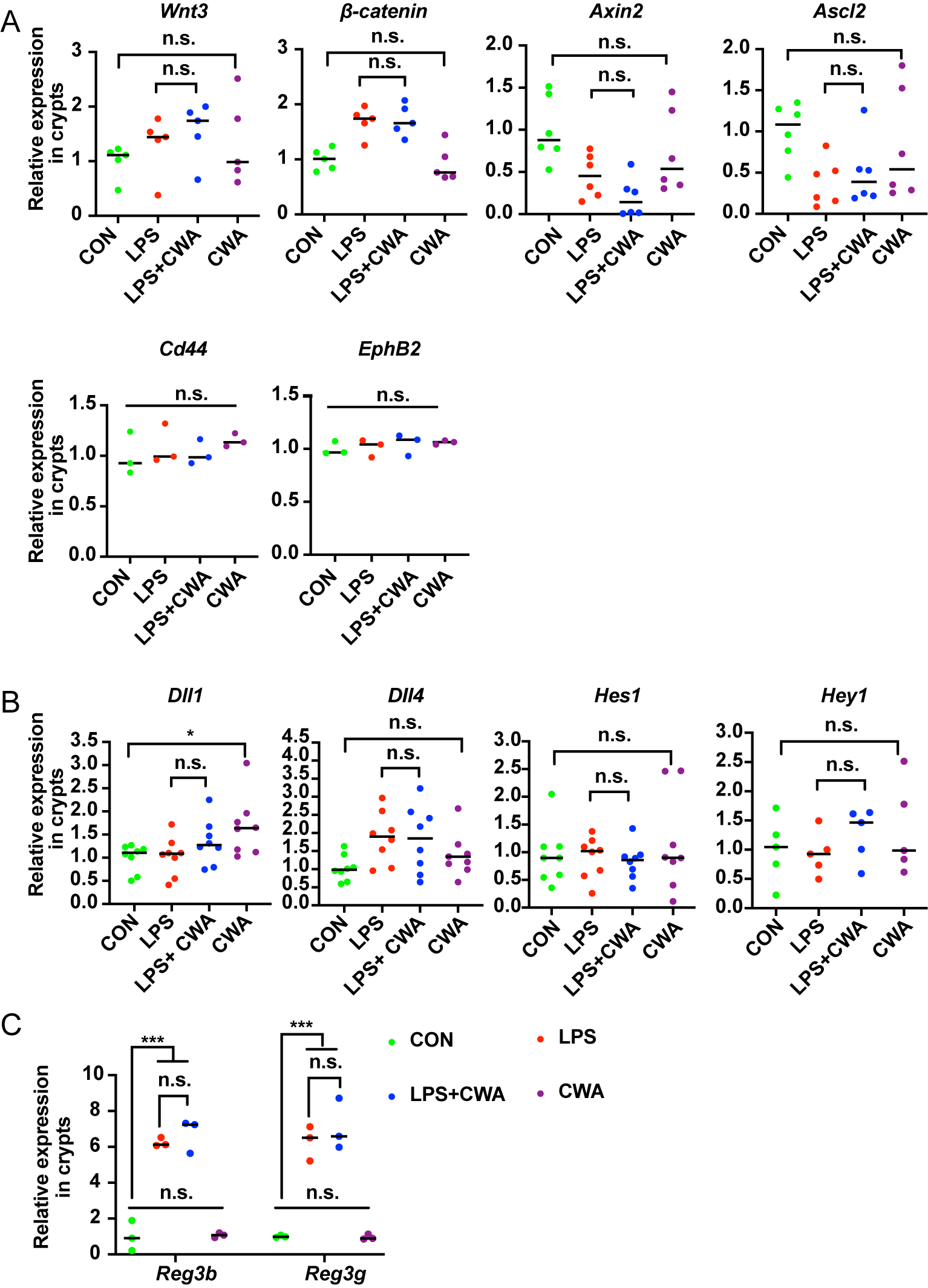

Supplement: Supplementary Figure 3 — CWA promotes intestinal epithelial proliferation independent of Notch, Wnt/β-catenin signaling pathways and innate AMPs. (A) RT-qPCR analysis of relative crypts mRNA expression of Wnt pathway genes in crypts. (B) RT-qPCR analysis of relative crypts mRNA expression of Notch pathway genes in crypts. (C) RT-qPCR analysis for relative crypts mRNA expression of Reg3b and Reg3g innate antimicrobials in crypts. ∗∗∗P < 0.001 by two-sided, unpaired t-test. All data represent at least three independent experiments. [file Image_3.TIF]

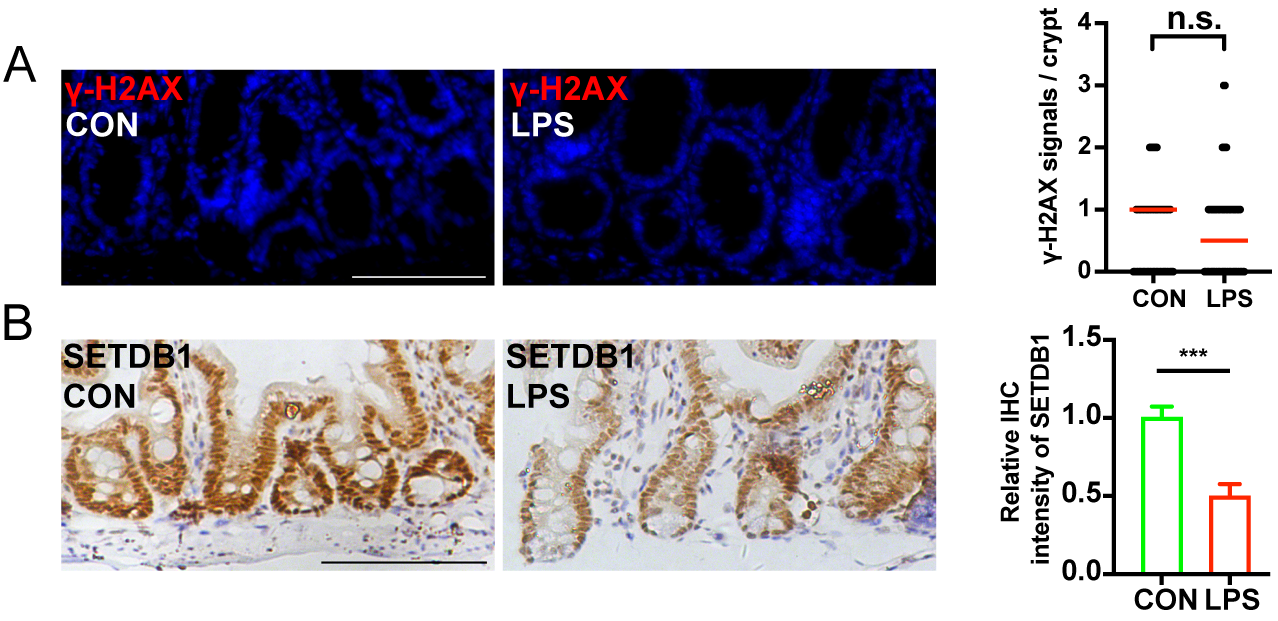

Supplement: Supplementary Figure 4 — Immunofluorescence and IHC analysis of γ-H2AX and SETDB1. (A) Representative immunofluorescence images of γ-H2AX expression in crypt at 6 h after LPS injection and numbers of γ-H2AX+ cells in crypts at 6 h after LPS injection. (B) Representative IHC image of SETDB1 expression at 6 h after LPS injection and analysis of SETDB1 IHC intensity. ∗∗∗P < 0.001 by two-sided, unpaired t-test. All data represent at least three independent experiments. [file Image_4.TIF]
